# Supplementary material for: Structural insights into the light-driven auto-assembly process of the water-oxidizing Mn4CaO5-cluster in photosystem II
Source: eLife. 2017 Jul 18;6:e26933. doi: 10.7554/eLife.26933 (PMC5542773; doi:10.7554/eLife.26933)
Supplement: Supplementary file 1. — The oxygen evolution was assayed at 25°C using different concentrations of NH2OH from 0 mM to 2 mM. *The rates are the averages of at least three repeated measurements. †The percentage of control rates are given in parentheses. DOI: http://dx.doi.org/10.7554/eLife.26933.019 [file elife-26933-supp1.docx]

**Supplementary File 1.** Effect of NH_2_OH on inhibition of oxygen evolution rates in intact C_12_E_8_-dPSIIcc.

| Concentration of NH_2_OH [mM] | [µmol of O_2_ h^-1^ (mg of Chl)^-1^]* activity after dilution and 30 s incubation with different amounts of NH_2_OH |
| --- | --- |
|  | C_12_E_8_ dPSIIcc (%)^†^ |
| 0 | 2669±454 (100) |
| 0.01 | 1395±58 (52) |
| 0.05 | 1073±26 (40) |
| 0.1 | 945±20 (35) |
| 0.5 | 732±34 (27) |
| 1 | 325±35 (12) |
| 2 | 137±98 (5) |

*The rates are the averages of at least three repeated measurements.

^†^The percentage of control rates are given in parentheses.
