# Supplementary material for: Structural insights into the light-driven auto-assembly process of the water-oxidizing Mn4CaO5-cluster in photosystem II
Source: eLife. 2017 Jul 18;6:e26933. doi: 10.7554/eLife.26933 (PMC5542773; doi:10.7554/eLife.26933)
Supplement: Supplementary file 2. — ‡ n-Dodecyl ß-D-maltoside Masses from linear mode MALDI-TOF-MS and assigned to PSIIcc subunits. The experimental determined mass from the C12E8 dPSIIcc treated with 50 mM NH2OH/EDTA is the average mass from spectra recorded from three independent sample preparations. n.d.: not detectable DOI: http://dx.doi.org/10.7554/eLife.26933.020 [file elife-26933-supp2.docx]

**Supplementary File 2.** Completeness of PSII subunits after NH_2_OH/EDTA treatment.

|  |  | **C_12_E_8_-dPSIIcc standard** | **with 50 mM NH_2_OH/EDTA treated C_12_E_8_-dPSIIcc** | **ß-DM** ‡ **dPSIIcc standard** |
| --- | --- | --- | --- | --- |
| Subunit | cyanobase number | experimental determined masses in m/z [M+H]^+^ in Da | experimental determined masses in m/z [M+H]^+^ in Da | experimental determined masses in m/z [M+H]^+^±$\sigma$ |
| PsbT | tsr1531 | 3896.6 | 3903.9 | 3906±4 |
| PsbM | tsl2052 | 3938.6 | 3937.8 | 4011±2 |
| PsbJ | tsr1544 | 4014.0 | 4026.9 | 4017±2 |
| PsbK | tsl0176 | 4101.8 | 4101.1 | 4103±4 |
| PsbX | tsr2013 | 4190.7 | 4190.2 | 4192±4 |
| PsbL | tsr1543 | 4300.0 | 4307.0 | 4301±4 |
| PsbI | tsr1074 | 4436.2 | 4443.6 | 4437±4 |
| PsbY | tsl0836 | 4616.6 | 4631.8 | 4617±4 |
| PsbF | tsr1542 | 4979.3 | 4978.8 | 4981±5 |
| ycf12 | tsr1242 | 5068.8 | 5084.4 | 5068±2 |
| PsbZ | tsr1967 | 6797.5 | 6804.9 | 6798±5 |
| PsbH | tsl1386 | 7205.1 | 7204.7 | 7227±5 |
| PsbE | tsr1541 | 9445.1 | 9445.0 | 9446±6 |
| PsbU | tll2409 | 11645.8 | n.d. | 11649±8 |
| PsV | tll1285 | 15747.7 |  | 15752±11 |
| PsbO | tll0444 | 26814.0 |  | 26830±30 |

‡ n-Dodecyl ß-D-maltoside

Masses from linear mode MALDI-TOF-MS and assigned to PSIIcc subunits. The experimental determined mass from the C_12_E_8_ dPSIIcc treated with 50 mM NH_2_OH/EDTA is the average mass from spectra recorded from three independent sample preparations.

n.d.: not detectable
